# How Sex and Race/Ethnicity Influence Interventional Cardiology Outcomes: What Do We Know?

**DOI:** 10.1016/j.jscai.2026.104262

**Published:** 2026-02-12

**Authors:** Niki Procopi, Francesca Maria Di Muro, Birgit Vogel, Roxana Mehran

**Affiliations:** a Center for Interventional Cardiovascular Research and Clinical Trials, Icahn School of Medicine at Mount Sinai, New York, New York; b Sorbonne Université, Hôpital Pitié-Salpêtrière (AP-HP), ACTION Study Group, INSERM UMRS 1166, Institut de Cardiologie, Paris, France

**Keywords:** ethnicity, interventional cardiology, outcomes, percutaneous coronary intervention, race, sex

## Abstract

Despite substantial advances in interventional cardiology, profound disparities in access to care and clinical outcomes remain across racial/ethnic and sex groups. These inequities arise from a complex interplay of biological differences, structural barriers, and sociocultural determinants that shape disease recognition, treatment allocation, procedural decision-making, and long-term follow-up. A further driver is the limited understanding of cardiovascular disease in female patients and racial/ethnical minorities, largely attributable to their persistent underrepresentation in clinical research, thereby restricting the external validity of evidence and perpetuating gaps in practice. This review explores how social and biological dimensions intersect to influence risk profiles, clinical presentation, procedural strategies, and secondary prevention in contemporary practice, focusing on the 2 most widely performed interventions in cardiology: percutaneous coronary intervention and transcatheter aortic valve replacement. It also highlights how such differences are often not integrated into standardized pathways of care, allowing inequities to persist even in the era of advanced cardiovascular technologies. Ultimately, it may serve as a call to action: advancing equity in interventional cardiology requires more than representative trial enrollment. It demands reform in study design, systematic stratification of outcomes, and structural change in care delivery to ensure that innovations in cardiovascular medicine benefit all patients.

## Introduction

Cardiovascular disease remains the leading cause of death in high income countries, yet progress in prevention and treatment has been uneven. Coronary artery disease (CAD) continues to drive this burden, with persistent and widening disparities across sex and race/ethnicity.^1^ Despite major advances in interventional cardiology and substantial reductions in overall CAD mortality, these gains have not extended to all populations: mortality is rising among young women aged 35 to 54 years, and racial/ethnic minorities continue to experience disproportionately worse outcomes.^2^, ^3^, ^4^, ^5^, ^6^ Percutaneous coronary intervention (PCI) is central-to-contemporary CAD management, with more than 600,000 procedures performed annually in the United States. However, only one-third involve female patients, and roughly one-fifth involve patients from racial/ethnic minority groups.^7^, ^8^, ^9^ These same populations remain markedly underrepresented in randomized controlled trials (RCTs), limiting the external validity of contemporary evidence and potentially reinforcing inequities in care delivery and clinical outcomes.^10^ Sex and race/ethnicity capture distinct but intersecting dimensions of cardiovascular risk. Sex reflects biological and hormonal differences as well as gendered social exposures, whereas race/ethnicity—although not a biological category—functions as a proxy for structural and social determinants that shape access, treatment patterns, and long-term outcomes. Emerging evidence shows that these factors independently—and synergistically—affect disease presentation, procedural pathways, and postintervention prognosis. Despite increasing recognition of these gaps, a consolidated, cross-procedural synthesis is still missing. This review synthesizes contemporary evidence on sex- and race/ethnicity-related disparities across the 2 predominant cardiac interventions—PCI and transcatheter aortic valve replacement (TAVR).

## Sex disparities in interventional cardiology

### Sex-related differences in characteristics and outcomes in the population undergoing PCI

Women undergoing PCI typically present with a higher cardiovascular risk profile than men. They are, on average, older at the time of intervention (around 66.0 vs 61.4 years) and exhibit a greater prevalence of comorbidities, such as diabetes mellitus, hypertension, and hyperlipidemia.^11^, ^12^, ^13^ Beyond the traditional risk factors, female patients are also affected by sex-specific cardiovascular risk factors, including premature menopause, hypertensive disorders of pregnancy, preterm delivery, and gestational diabetes.^14^^,^^15^ Sex-based differences also extend to coronary anatomy. Female patients generally have smaller vessel diameters and higher vascular tortuosity than male patients, features associated with altered shear stress conditions that may promote atherosclerosis development.^16^, ^17^, ^18^ Differences in plaque morphology have likewise been observed: women show an age-related increase in rupture-prone phenotypes—such as lipid-rich plaques, thin-cap fibroatheroma, and inflammatory microstructures—whereas no similar trend is noted in men.^19^ Clinical presentation of CAD varies by sex, with female patients being more likely to present with unstable angina and less frequently with acute coronary syndromes (ACS) than male patients.^16^^,^^20^^,^^21^ Furthermore, compared with obstructive CAD, female patients more frequently manifest nonobstructive CAD and coronary microvascular dysfunction, accounting for approximately two-thirds of cases, which have been linked with an increased risk of adverse cardiovascular outcomes.^22^, ^23^, ^24^

Although findings vary across clinical presentations, a growing body of evidence suggests that female patients undergoing PCI experience worse outcomes than male patients.^25^ In a pooled analysis of 11 ACS trials conducted between 1993 and 2006, the overall 30-day mortality was similar between sexes; however, a significant difference emerged according to ACS subtype. After adjustment for cardiovascular risk factors, the 30-day mortality was higher among female patients with ST-elevation myocardial infarction (STEMI), whereas no excess risk was observed in non-STEMI or unstable angina.^26^ Similarly, a pooled analysis of 21 PCI RCTs including 32,877 patients demonstrated a higher 5-year risk of major adverse cardiac events (MACE), all-cause mortality, and cardiac mortality in female patients compared with male patients (18.9% vs 17.7%; *P* = .003 for MACE).^16^ These findings are consistently confirmed when moving from trial populations to real-world settings. A large meta-analysis of 35 observational studies in STEMI patients treated with PCI demonstrated higher in-hospital mortality among female patients, potentially driven by early postprocedural complications.^27^ Similarly, in a nationwide US registry including approximately 6 million PCI procedures (Nationwide Inpatient Sample), in-hospital mortality was significantly higher in female patients than in male patients, corresponding to a ∼20% relative excess risk (OR, 1.20; 95% CI, 1.16-1.23).^21^ Bleeding and vascular complication rates are also consistently higher in female patients—particularly younger female patients—even after adjustment for confounders, with a 2-fold increase in major bleeding and a 1.5-fold increase in vascular complications.^20^^,^^21^

### Sex-related differences in TAVR population and outcomes

Transcatheter aortic valve replacement has become the second most commonly performed interventional cardiology procedure, after PCI. The proportion of female patients undergoing TAVR in studies and registries ranges from 44% to 58%, exceeding their representation among patients with CAD.^28^, ^29^, ^30^, ^31^, ^32^ Female patients undergoing TAVR have a distinct risk profile and procedural characteristics compared with male patients, underscoring the need to study female patients as a distinct population.^33^ Female patients presenting for TAVR are typically older, have higher surgical risk scores, but fewer traditional atherosclerotic comorbidities such as CAD and diabetes compared with male patients.^31^^,^^34^ Female patients more often have peripheral vessels and smaller annuli, which substantially influence the valve selection because of an increased risk of prosthesis-patient mismatch.^28^ This is a good example of a condition that has been specifically addressed in an RCT mostly involving female patients with small aortic annuli, which demonstrated that a self-expanding supraannular valve was superior to a balloon-expandable valve in reducing bioprosthetic valve dysfunction at 12 months.^35^^,^^36^ Female patients experience higher rates of vascular complications and major or life-threatening bleeding, as well as increased short-term (30-day) mortality and stroke, particularly in the oldest age groups.^31^^,^^37^, ^38^, ^39^, ^40^, ^41^, ^42^, ^43^, ^44^ Despite these early risks, female patients often demonstrate similar or lower 1-year mortality compared with male patients after TAVR,^44^ and also have lower rates of acute kidney injury and permanent pacemaker implantation post-TAVR.^45^^,^^46^

## Racial/ethnic disparities in interventional cardiology

### Definition and representation of race/ethnicity

Race/ethnicity is a multifaceted concept encompassing genetic background linked to geographic ancestry, as well as cultural, environmental, and socioeconomic factors.^47^ The US population is racially/ethnically diverse and expected to become even more so. Among minority groups, higher levels of acculturation (eg, US-born, longer US residence, English language preference) are associated with a greater burden of cardiovascular risk factors, especially within Hispanic and Asian populations, which illustrates the complex interplay between exposome, encompassing environment, culture, and lifestyle and genetic factors in shaping cardiovascular health.^48^^,^^49^ Currently, approximately 61% of the US population identifies as non-Hispanic White, 19% as Hispanic, 13% Black, 6% Asian, and 1% American Indian. By 2060, non-Hispanic White individuals are projected to decrease from 62.2% in 2014 to 43.6%, Asians to rise from 5.4% to 9.3%, Black Americans to increase slightly from 13% to 14%, and the multiracial population is expected to grow from 2.5% to 6.2%.^50^ Most trials rely on self-reported race/ethnicity, but classifications vary by study and region, complicating assessment of racial/ethnic-specific outcomes. Moreover, they do not systematically further stratify subgroups within broader categories—for example, they do not distinguish between South Asian and East Asian individuals, despite their markedly different cardiovascular risk profiles and post-PCI outcomes.

The largest RCTs on cardiac interventions, forming current guidelines, have predominantly enrolled patients from North America, Europe, and East Asia.^6^^,^^51^, ^52^, ^53^, ^54^, ^55^, ^56^, ^57^, ^58^, ^59^, ^60^, ^61^, ^62^, ^63^, ^64^, ^65^, ^66^, ^67^, ^68^ Many do not specify race/ethnicity, probably because of data collection regulations or low diversity. A pooled analysis of 22,638 patients from 10 PCI RCTs assessed differences in risk factors, comorbidities, and clinical outcomes across racial/ethnic groups, using non-Hispanic White as the reference.^69^ Consistent with previous findings, the racial/ethnic distribution of enrolled patients did not reflect the US population, as only 8% were from minority groups.^70^ To address this underrepresentation, a monocentric registry of patients undergoing PCI between 2012 and 2022 at The Mount Sinai Hospital, a high-volume quaternary care center in New York City, analyzed racial/ethnic differences in clinical characteristics and outcomes.^71^ Baseline variables from both data sets are summarized in Tables 1 and 2.^69^^,^^71^

**Table 1:** Clinical characteristics of patients after undergoing PCI by ethnic group: evidence from RCTs and real-world clinical practice.

Characteristics	White		Black		Hispanic		Asian		
	In RCT^a^ (n = 20,585)	Registry Mount Sinai^b^ (n = 10,161)	In RCT^a^ (n = 918)	Registry Mount Sinai^b^ (n = 2390)	In RCT^a^ (n = 473)	Registry Mount Sinai^b^ (n = 3828)	In RCT^a^ (n = 404)	Registry Mount Sinai^b^	
								South Asian (n = 4369)	East Asian (n = 458)
Proportion	92.0 (20,585/22,380)	47.6 (10,161/21,236)	4.1 (918/22,380)	11.6 (2390/21,236)	2.1 (473/22,380)	18.0 (3828/21,236)	1.8 (404/22,380)	21.8 (4369/21,236)	2.3 (458/21,236)
Age, y	62.8 ± 11.1	69.0 ± 11.7	59.4 ± 11.2	63.9 ± 11.7	62.0 ± 11.2	66.5 ± 11.2	62.0 ± 11.2	62.1 ±10.1	65.9 ± 12.1
Women	27.4 (5640/20,585)	24.1 (2290/9520)	42.4 (389/918)	45.9 (1061/2313)	36.8 (174/473)	37.9 (1383/3653)	23.3 (94/404)	21.0 (918/4369)	24.3 (111/457)
Body mass index, kg/m^2^	29.6 ± 5.7	29.2 ± 5.7	31.7 ± 6.7	30.1 ± 6.5	30.3 ± 6.2	29.1 ± 5.6	25.5 ± 4.3	27.0 ± 4.5	25.6 ± 4.2
Diabetes mellitus	25.0 (5140/20,529)	35.5 (3375/9520)	42.7 (391/916)	53.0 (1225/2313)	47.4 (224/473)	56.1 (2048/3653)	29.2 (118/404)	62.7 (2738/4369)	49.5 (226/457)
Current smoking	28.5 (5811/20,365)	11.9 (1133/9520)	33.3 (304/912)	19.3 (447/2313)	21.5 (101/469)	12.9 (471/3653)	20.5 (82/400)	10.8 (473/3369)	7.7 (35/457)
Hypertension	67.4 (13,844/20,527)	89.5 (8521/9520)	84.6 (775/916)	94.3 (2185/2313)	75.9 (359/473)	93.7 (3423/3653)	67.3 (272/404)	94.1 (4111/4369)	89.9 (411/457)
Hyperlipidemia	64.2 (13,070/20,344)	89.7 (8536/9520)	69.0 (624/905)	87.3 (2020/2313)	65.3 (307/470)	87.3 (2020/2313)	73.8 (295/400)	93.8 (4099/4369)	88.8 (406/457)
Clinical presentation									
Acute coronary syndrome	68.1 (13,076/19,199)	40.3 (3834/9520)	67.8 (566/835)	48.7 (1126/2313)	83.5 (386/462)	42.4 (1547/3653)	49.7 (183/368)	32.4 (1414/4369)	40.2 (184/457)
STEMI	14.8 (2837/19,199)	2.7 (261/9520)	5.6 (47/835)	5.1 (117/2313)	0	4.8 (174/3653)	5.2 (19/368)	1.8 (79/4369)	3.9 (18/457)
NSTEMI	23.0 (4412/19,199)	12.6 (1196/9520)	23.1 (193/835)	17.3 (401/2313)	37.9 (175/462)	11.9 (436/3653)	16.0 (59/368)	8.7 (380/4369)	12.7 (58/457)
Unstable angina	30.4 (5827/19,1990	25.0 (2377/9520)	39.0 (326/835)	26.3 (608/2313)	45.7 (211/462)	25.7 (937/3653)	28.5 (105/368)	21.9 (955/4369)	23.6 (108/457)
Stable CAD	31.9 (6123/19,199)	58.2 (5543/9520)	32.2 (269/835)	49.8 (1152/2313)	16.5 (76/462)	57.1 (2085/3653)	50.3 (185/368)	66.5 (2906/4369)	58.4 (267/457)

Values are mean ± SD or % (n/N).

CAD, coronary artery disease; NSTEMI, non–ST-elevation myocardial infarction; PCI, percutaneous coronary intervention; RCT, randomized controlled trial; STEMI, ST-elevation myocardial infarction.

a From Golomb et al.^69^

b From Kapoor et al.^71^

**Table 2:** One-year outcomes after PCI across race/ethnic groups: Evidence from RCTs and real-world clinical practice.

Outcomes	White		Black				Hispanic				Asian					
	In RCT^a^	Registry Mount Sinai^b^	In RCT^a^		Registry Mount Sinai^b^		In RCT^a^		Registry Mount Sinai^b^		In RCT^a^		Registry Mount Sinai^b^			
	n = 20,585	n = 10,161	n = 918	*P* value^c^	n = 2390	Adjusted *P* value^c^	n = 473	*P* value^c^	n = 3828	Adjusted P value^c^	n = 404	*P* value^c^	South Asian		East Asian	
													n = 4399	Adjusted *P* value^c^	n = 458	Adjusted *P* value^c^
All-cause death	2.2 (445)	3.3 (296)	3.2 (28)	.060	4.6 (93)	.007	2.6 (12)	.53	2.8 (93)	.039	1.3 (5)	.21	1.7 (63)	<.001	4.5 (17)	.615
MI	4.9 (990)	2.2 (186)	7.2 (65)	.001	3.0 (60)	.913	7.5 (35)	.008	2.5 (82)	.123	2.8 (11)	.052	1.8 (67)	.014	0.9 (3)	.054
MACE	10.4 (2106)	5.4 (472)	13.1 (117)	.008	7.4 (151)	.044	13.3 (61)	.04	5.5 (181)	.052	6.8 (27)	.02	3.3 (125)	<.001	5.7 (21)	.642

Values are % (n/N). Time-to-event variables are summarized by Kaplan-Meier event rates % (number of events). The White population was used as the reference value.

MACE, major adverse cardiovascular events; MI, myocardial infarction; PCI, percutaneous coronary intervention; RCT, randomized controlled trial.

a From Golomb et al.^69^

b From Kapoor et al.^71^

c Reference = White.

### Black vs White patients undergoing PCI

Compared with White patients, Black patients with CAD more often have hypertension, diabetes, and obesity, are younger, more frequently female patients, and have a lower socioeconomic position, but do not differ in dyslipidemia or smoking.^59^^,^^69^^,^^72^, ^73^, ^74^, ^75^, ^76^, ^77^, ^78^ Despite this high-risk profile, Black patients often have less extensive and complex CAD than White patients, with fewer left main or left anterior descending artery disease, shorter lesions, fewer bifurcations, and less multivessel disease.^69^^,^^71^^,^^74^^,^^79^^,^^80^ They also tend to have similar or lower coronary artery calcium scores,^81^, ^82^, ^83^, ^84^, ^85^ suggesting that anatomic severity does not explain outcome disparities. Clinical presentation data are conflicting: RCTs indicate that Black patients present less often with STEMI and more with unstable angina, whereas registries report fewer chronic presentations and more ACS.^69^^,^^71^^,^^86^ Outcome data are inconsistent: a medium-sized prospective multicenter registry found no difference in 1-year mortality between Black and White patients,^74^ whereas a large pooled analysis of RCTs reported higher rates of 1-year all-cause death and myocardial infarction (MI) following PCI in Black patients compared with White patients. At 5 years, mortality was similar, but MI and MACE were significantly higher in Black patients.^69^ A retrospective cohort from a high-volume New York center similarly showed a higher 1-year mortality in Black patients, even after adjusting for comorbidities and socioeconomic status (SES).^71^^,^^87^ In another large registry from Duke University, 15-year mortality was also higher in Black patients (35.5% vs 45.1%), particularly in Black female patients (30.7%), persistently higher after adjustment for comorbidities, socioeconomic factors, and treatment.^77^

### Hispanic vs non-Hispanic White patients undergoing PCI

Compared with non-Hispanic White patients, Hispanic patients with CAD have higher rates of hypertension, diabetes, and obesity, and are more frequently women, while age, smoking, and dyslipidemia are similar.^69^ Despite a higher risk profile, 30-day, 1-year, and 5-year all-cause mortality rates in Hispanic patients enrolled in coronary intervention RCTs were similar to or lower than non-Hispanic Whites.^69^^,^^71^ Furthermore, Hispanic patients exhibited less involvement of the left main or left anterior descending artery, fewer bifurcations, and shorter coronary lesions.^71^ This discrepancy between a higher cardiovascular risk profile but lower event rates in Hispanic individuals compared with White individuals, known as the “Hispanic paradox,” may be linked to genetic factors.^88^

### Asian patients vs non-Hispanic White patients undergoing PCI

Substantial heterogeneity has been documented within the Asian population with CAD, emphasizing the need to distinguish South Asian from East Asian patients, though few studies do so.^71^^,^^89^ Compared with White patients, South Asian patients undergoing PCI are younger, more often of male sex, and have a higher prevalence of diabetes, hypertension, and dyslipidemia, with similar smoking rates, and a lower body mass index. East Asian patients undergoing PCI are younger and more frequently of male sex, have lower smoking rates, a higher prevalence of diabetes, lower body mass index, and similar rates of hypertension and dyslipidemia compared with White patients.^71^ Asian patients—especially those from South Asia—are more likely to present with stable CAD (approximately 50% doing vs 32% in White patients) and show lower all-cause mortality at 1 year and during long-term follow-up.^69^^,^^71^^,^^90^ These differences do not appear to be attributable to coronary lesion severity,^71^ or by the underlying mechanisms of ACS, which appear to be similar based on optical coherence tomography findings.^91^

### Native-American patients vs non-Hispanic White patients undergoing PCI

Data on Native-American patients undergoing PCI are limited. Native Americans tend to develop CAD at a younger age, on average 5 years earlier than White Americans—probably because of a markedly higher prevalence of diabetes (24% vs 8%).^92^ They also exhibit higher incidence and mortality rates from CAD, particularly at younger ages. Although in-hospital mortality from ACS has declined across all racial/ethnic groups, the pace of this improvement has been slower among Native Americans.^93^^,^^94^

### Ethnic/racial disparities in the population undergoing TAVR

Minority populations are largely underrepresented in US TAVR registries.^95^ Black, Hispanic, and Asian patients undergoing TAVR present with a higher burden of comorbidities such as diabetes, chronic kidney disease, and heart failure compared with their White counterparts.^37^^,^^96^ Anatomic differences have been reported, with Asians tending to show smaller annular dimensions and vascular access, which can influence procedural planning.^96^ Outcomes following TAVR are broadly comparable across racial and ethnic groups after adjustment for comorbidities and procedural characteristics, despite the persistent underrepresentation.^95^^,^^97^, ^98^, ^99^, ^100^

## Socioeconomic disparities in interventional cardiology

Socioeconomic status—encompassing income, education, employment, neighborhood environment, and acculturation—shapes cardiovascular risk, symptom recognition, access to care, and postprocedural outcomes. Because SES intersects with both sex and race/ethnicity, isolating its independent effect is challenging, yet evidence consistently shows that SES is a major structural determinant of interventional cardiology care.^101^ The Centers for Disease Control and Prevention’s social vulnerability index (SVI) is increasingly used to quantify community-level socioeconomic vulnerability. Although its application in interventional cardiology remains limited, available data show that patients living in high-SVI counties show lower rates of referral for PCI, experience greater delays in revascularization, and show worse postprocedural outcomes compared with those in low-SVI counties, even after adjustment for comorbidities and clinical presentation.^102^^,^^103^

Low SES exerts a disproportionately negative impact on cardiovascular health in female patients compared with male patients.^104^ Female patients with lower SES face multiple, compounding barriers across the care continuum. They are less likely to receive cardiology consultations for CAD and substantially less likely to be referred to cardiac rehabilitation at discharge, despite strong mortality reductions in female patients who participate.^105^^,^^106^ These disparities likely reflect a combination of caregiver responsibilities, lower health literacy, systemic bias, and reduced access to resources that enable follow-up care. Pharmacologic management is also affected. High-risk female patients are more frequently prescribed ezetimibe and PCSK9 inhibitors than male patients, a pattern driven by higher rates of uncontrolled dyslipidemia and statin intolerance.^107^^,^^108^ Paradoxically, female patients remain less likely to receive antiplatelet agents and statins for long-term secondary prevention,^109^ and disparities in preventive therapy persist even among those with insurance. Sex-specific differences in platelet biology and bleeding risk complicate antithrombotic management,^110^^,^^111^ though randomized evidence—including TWILIGHT—demonstrates that early aspirin discontinuation and ticagrelor monotherapy are equally effective and safe in female patients and male patients.^112^^,^^113^ In acute settings, socioeconomic and structural barriers amplify sex disparities. Female patients presenting with STEMI and cardiogenic shock are older, have more comorbidities, undergo fewer invasive procedures (including revascularization, right heart catheterization, and mechanical circulatory support), and experience significantly higher in-hospital mortality (40%-45%) compared with male patients (30%-35%).^114^ These inequities reflect delays in triage, underrecognition of symptoms, and differential treatment pathways—mechanisms closely linked to SES.

Socioeconomic disadvantage is also a central driver of racial/ethnic disparities in interventional cardiology. Black and Hispanic patients are less likely to receive cardiology consultations compared with care for White patients,^43^^,^^115^ and socioeconomic barriers—including limited specialist availability in underserved areas—appear to play a significant role. Pharmacologic inequities mirror these patterns. Non-Hispanic Black and Asian patients are less likely to receive ezetimibe or PCSK9 inhibitors after MI, limiting their ability to reach LDL-C targets below 55 mg/dL.^116^ Long-term adherence to statins and antiplatelet therapy is lower among Black and Hispanic male patients compared with White male patients,^117^ and disparities persist even after accounting for insurance status. Cardiac rehabilitation remains markedly underutilized among Black, Hispanic, and Asian patients, with consistently lower referral rates at discharge.^2^

Procedural disparities highlight how SES intersects with systemic factors to shape interventional care. Black, Hispanic, and Asian patients with ACS experience longer door-to-balloon times, lower PCI utilization, and differential management of heart attack, potentially leading to worse outcomes, compared with White patients.^116^^,^^118^, ^119^, ^120^ For TAVR, procedure rates are lower in regions with higher proportions of minority individuals—even after adjusting for disease burden—while large registries show that Black, Hispanic, and Asian patients are less likely to be referred for evaluation of aortic stenosis.^115^^,^^117^^,^^121^

### How can clinical trials mobilize diversity efforts?

Overall, we observe sex and racial/ethnic disparities in patient profiles and outcomes, as summarized in the Central Illustration. In response, leading cardiovascular societies have long advocated for more inclusive research, culturally competent care, and equitable implementation of guideline-directed therapies, which led to the National Institutes of Health Revitalization Act >30 years ago, mandating the inclusion of women and underrepresented groups in clinical research; nonetheless, the representation of women in cardiovascular research remains inadequate.^122^, ^123^, ^124^, ^125^ It requires structural changes at every stage—from design to publication.^126^ Adaptive trial designs offer an interesting approach by allowing real-time modifications to enrollment criteria based on interim data, improving the representativeness of study populations. Enrolling patients from historically underrepresented communities in RCTs requires targeted and often challenging efforts to broaden recruitment strategies. Trial sponsors and funders should encourage diversity benchmarks and enforce accountability mechanisms to ensure these goals are met. Programs such as the ACC Clinical Trials Research program also support these efforts by training cardiovascular professionals in designing and leading trials, fostering a diverse and skilled research workforce.

**Central Illustration:**
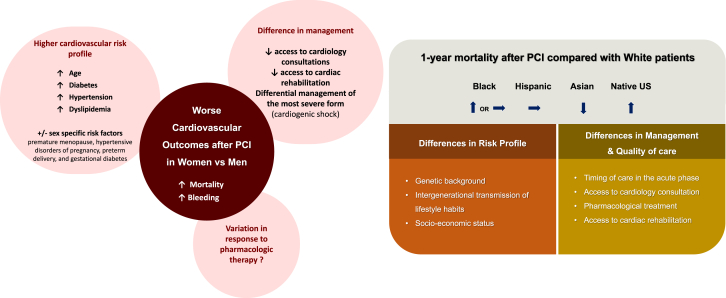
**Determinants of outcome differences after percutaneous coronary intervention (PCI) across racial/ethnic groups (vs non-Hispanic White) and between female and male patients.**

Leadership diversity is equally important, as trials led by female patients and individuals from minority groups are more likely to enroll diverse participants.^126^, ^127^, ^128^ Institutional initiatives should promote inclusive leadership through structured mentorship, equitable advancement opportunities, and active efforts to counter implicit bias. Creating harassment-free, respectful environments and using inclusive, nonstereotyped language are essential to building trust within research teams and participant populations.

Finally, consistent and transparent reporting of participant demographics—free from stereotyping—is essential to monitor progress and guide clinical practice. By embedding these principles into trial design and execution, researchers can generate more generalizable evidence, support precision medicine, and ultimately improve health outcomes for all populations.

## Conclusion

Sex and race/ethnicity are independent—and often intersecting—determinants of outcomes in interventional cardiology. Female patients remain at increased risk of both ischemic and bleeding events after PCI, whereas racial/ethnic minorities—particularly Black patients—experience higher rates of adverse outcomes, even after adjustment for baseline and socioeconomic factors. These disparities are shaped by a complex interplay of biological, structural, and systemic contributors, including underrecognition of disease, unequal access to care, and differences in the use of evidence-based therapies, despite decades of advancements in procedural techniques and pharmacologic strategies. Addressing these inequities requires a shift from observation to implementation: inclusive trial designs, systematic collection of sex- and race/ethnicity-specific data, and proactive integration of equity-focused care models into clinical practice. Ensuring that interventional advances benefit all populations equitably is not only a matter of scientific rigor but of social responsibility.
